# The ADHD debate: being mindful of complexity and wary of reductionist explanations and polarization Commentary on 'A social relational critique of the biomedical definition and treatment of ADHD; ethical, practical and political implications'

**DOI:** 10.1111/j.1467-6427.2012.00608.x

**Published:** 2012-11-01

**Authors:** Anita Thapar, Kate Langley, Antonio Muñoz-Solomando

**Affiliations:** a Section of Child and Adolescent Psychiatry, Institute of Psychological & Clinical Neurosciences, School of Medicine, Cardiff University; b Research Associate Psychologist; 3 Tonteg Child and Family Clinic, Tonteg HospitalChurch Road, Pontypridd, CF38 1HE, UK

## Abstract

The editor of this journal has asked for our opinion on the article by Wilson (2012). In the interests of open dialogue we have agreed. We do not intend to critique the article in detail or to select specific sentences that we agree or disagree with. Our aim is to highlight what we consider important relevant issues.

## Is it ethical to practice what we personally like and believe in, according to our tradition and professional background?

First, let us make it clear that we agree debate and discussion are important. We also believe that when examining questions of significant social, clinical, practice and policy relevance it is important to start with a hypothesis. As observed by a family therapist, it is equally important ‘not to fall in love’ with our hypotheses (Rivett, 2012) and to test them rigorously. Our view is that hypotheses need to be empirically tested and challenged. This makes the difference between pronouncements that reflect personal views or professional preferences and those that reflect evidence. It is our opinion and that of many others that it is unethical and at times dangerous to attempt to help people according to our personal views and professional preferences. For those of us who are employed as public sector employees, where families are referred to us, our task is to provide help that has been shown (not by us necessarily) to be effective and that is feasible and pragmatic.

## The dichotomization of biological/medical and non-biological/non-medical is unhelpful and incorrect

It is not completely clear to us whether the author is or is not attempting to dichotomize explanations and conceptualizations that are genetic/biological/medical and those that are non-biological/non-medical. We wish to emphasize that dichotomization is not only unhelpful but also factually incorrect. Firstly, the social/non-biological and biological cannot be separated (Rutter, 2007). Psychosocial adversity can result in biological and brain changes (Hackman *et al.,* 2010; Meaney and Szyf, 2005). Such changes can in turn result in or exacerbate social adversity or psychosocial stressors. Studies of risk factors, including genetic ones, show that most mental health problems and normal forms of behaviour are a complex mix of inherited and non-inherited factors that co-act and interact in a dynamic fashion (Thapar *et al.,* 2007). Many genetically sensitive, longitudinal, experimental and treatment studies (Ge *et al.,* 1996; Reid *et al.,* 2002) show evidence of circular or cyclical, not purely linear, causality. Secondly, the nature of interventions and the origins of a problem are separate. For example, phenylketonuria is caused by one faulty gene (as noted later, attention deficit hyperactivity disorder [ADHD] is more complex) but the treatment of choice is dietary intervention.

## Personal and professional preferences, when they prevent us from staying updated and from critically evaluating the literature and knowledge on topics we do not like or value, are potentially dangerous

The author highlights an important point. He mentions a father who believes 95 per cent of his son's ADHD is genetic. This interpretation is incorrect. ADHD is influenced by a complex mixture of genes, environment and their interplay that increase risk in a probabilistic fashion. There is no single cause and research findings are commonly misinterpreted and oversimplified. Presumably this father's information has come from reading heritability estimates of ADHD that do not mean 95 per cent of an individual's ADHD is genetic. For the purpose of this commentary we do not intend to provide detailed explanations of ADHD aetiology that can be found elsewhere (Taylor and Sonuga Barke, 2008; Thapar *et al.,* 2012). However the point is that, regardless of our personal interests and professional preferences, we need to stay updated to provide good quality information and explanation to families who might misinterpret evidence. For example, we might not be trained family therapists but when evidence emerges (Eisler and Lask, 2008) we need to update ourselves and ensure the effective new interventions are implemented. We might not like genetics but when evidence emerges we need to understand and appraise it so that we are able to communicate and clarify findings to families who ask.

## If we dispense with diagnostic labels including ADHD for those who are having serious problems, what are the alternatives?

We need methods that serve a useful purpose and have some sort of external validity (Wilcutt *et al.,* 2012). They need to allow practitioners to communicate with each other, their clients and the wider community and that will allow us to employ interventions that have been tested on people with similar presentations, not ones we necessarily prefer or are best trained in ourselves. That does not mean to say we should view people only within the context of their diagnostic terms. That leads us to our next point.

## We are not suggesting that evidence-based practice and the use of diagnosis means there is no place for common sense

Firstly, individuals who are given the same diagnosis (regardless of whether it relates to mental or physical health or both) are not the same. Diagnoses do not and should not define people. They are there as a tool. Just because people have the same diagnosis does not mean that they will be treated in the same way. Clinical and practitioner creativity and flexibility and tailoring the intervention to the individual and context are critical, as is user preference. Evidence-based approaches and national treatment and assessment guidelines do not mean we need to be unthinking. Secondly, diagnostic terms are not set in stone nor are they perfect representations of reality, and nor should they be revered (Rutter, 2011). They are a tool that provides a framework for evaluating evidence, applying it and communicating it. Diagnostic systems are flexible and have to be revised according to evidence, which means there will be change. Most mental health problems (such as ADHD and autism traits) and many physical health problems (for example, blood pressure/hypertension, blood glucose levels/diabetes) can be viewed as lying on a continuum. What is called disorder for these types of problems (unlike qualitatively distinct models of, for example, infectious disease) are extremes of continua that result in impairments and adverse consequences. The level of impairment will depend not only on the severity of the individual's characteristics but also on the interaction between it and the environment. If those with a propensity to diabetes were living in times of food shortage, this could confer advantage but that is not the case at present in high-income countries. To consider a medical model as purely innate/biological is an oversimplification.

## We need to be careful about being profession-centred. What about the families?

The ultimate aim of practitioners and researchers is to deliver help or establish evidence that, in the long run, helps individuals who are suffering or seeking help. Debate and critically evaluating practice and evidence are helpful but only as long as divided opinions and the polarization of team members does not result in nihilism, idiosyncratic practice or adverse effects on the well-being of those who seek our help. We must be careful where there is strong disagreement between clinical team members, even if these are unspoken, that this does not interfere with our engagement with families. We also need to listen to what children and their families say and take service user views into consideration.

## Conclusion

It is good to have dialogue and challenge current thinking but it is an academic luxury, in our view, to challenge without providing alternative empirically testable proposals. Where conceptualizations, personal beliefs and instinct-led interventions are offered to help families and children we need to be assured that such recommendations are evidence-based, pragmatic and affordable before services and practitioners might be expected to flexibly adapt to changes in evidence – irrespective of whether the evidence is incompatible with our personal beliefs or professional and practice preferences.
